# Auditory attentional selection is biased by reward cues

**DOI:** 10.1038/srep36989

**Published:** 2016-11-14

**Authors:** Erkin Asutay, Daniel Västfjäll

**Affiliations:** 1 Behavioral Sciences and Learning, Linköping University, SE - 581 83, Linköping, Sweden; 2 Civil and Environmental Engineering, Chalmers University of Technology, SE - 412 96, Gothenburg, Sweden; 3 Decision Research, 1201 Oak Street, Suite 200 Eugene, OR, USA.

## Abstract

Auditory attention theories suggest that humans are able to decompose the complex acoustic input into separate auditory streams, which then compete for attentional resources. How this attentional competition is influenced by motivational salience of sounds is, however, not well-understood. Here, we investigated whether a positive motivational value associated with sounds could bias the attentional selection in an auditory detection task. Participants went through a reward-learning period, where correct attentional selection of one stimulus (CS+) lead to higher rewards compared to another stimulus (CS−). We assessed the impact of reward-learning by comparing perceptual sensitivity before and after the learning period, when CS+ and CS− were presented as distractors for a different target. Performance decreased after reward-learning when CS+ was a distractor, while it increased when CS− was a distractor. Thus, the findings show that sounds that were associated with high rewards captures attention involuntarily. Additionally, when successful inhibition of a particular sound (CS−) was associated with high rewards then it became easier to ignore it. The current findings have important implications for the understanding of the organizing principles of auditory perception and provide, for the first time, clear behavioral evidence for reward-dependent attentional learning in the auditory domain in humans.

We receive a continuous stream of auditory input from our surroundings; and we are capable of processing this information flow, detect and identify salient objects around us, and attend to particular auditory events while pushing others into the background in a seemingly effective and effortless manner. Auditory attention theories suggest that the auditory system can decompose the complex acoustic input into separate streams of information^1^, which then compete for attentional selection to guide perception^2^,^3^. Attentional selection can be modulated by both stimulus-driven and goal-driven factors. Nevertheless, how emotional and motivational salience of stimuli influence attentional selection in the auditory domain is not well-understood. There is scant evidence on the impact of negative emotional salience of sounds on auditory attention (e.g. refs 4 and 5); however, attentional bias induced by positive stimuli in the auditory domain has not yet been investigated in humans. To remedy this, in the current study we studied how positive motivational value of stimuli modulates attentional selection in the auditory domain. We adopted a reward-learning paradigm to manipulate the motivational value of otherwise meaningless sounds to test their effect on auditory attention.

Previous research investigating the impact of rewards on attentional selection and perception employed reward-dependent attentional learning paradigms almost exclusively in the visual domain (e.g. refs 6, 7, 8, 9, 10). These studies typically involve an extensive training phase during which correct selection of certain visual features or objects are consistently associated with high-rewards while selection of other features are associated with low-rewards. The training phase is followed by a test phase during which the impact of reward-learning is evaluated. Critically, the test phase does not involve reward delivery. The results show that visual features that are associated with high rewards in the past can capture attention during the test phase involuntarily even when they are presented as task-irrelevant distractors. These findings suggest that learned stimulus-reward associations (i.e. value-learning) has an impact on attentional selection (i.e. value-driven attentional capture ref. 11). Additionally, it was found that if successful attentional suppression of a particular stimulus leads to high rewards in the past, then the same stimulus could be easily ignored during attentional selection in similar contexts^8^. Chelazzi and colleagues^12^ argued that the latter finding cannot be explained solely by learning simple stimulus-reward associations. Instead, they suggested that if participants believed that rewards depended on their performance, then learning would be an instrumental adaptation and influence specific attentional prioritization processes. Hence, learning-induced effects would be observed at the level of specific processes that led to rewards (i.e. target selection and distractor suppression). In the present study, we tested whether reward-dependent attentional learning could influence attentional selection and suppression mechanisms in the auditory domain.

The auditory system is involved in the detection of salient objects in our surroundings and has an orienting function for the organism^13^. Thus, it functions like an alarm system that scans for significant and relevant objects^14^. Since effective detection of rewarding and motivationally salient stimuli would be beneficial for the organism, we claim that auditory features could quickly gain motivational value through reward-learning, which in turn can bias attentional selection. To test this hypothesis, we designed an auditory attention task where participants located a target sound in the presence of a distractor. Hence, the task performance depended on successful selection of the target and successful suppression of the distractor. We, then, introduced a priori reward delivery schedule, where correct selection of a particular stimulus led to high reward while correct selection of another stimulus led to lower rewards.

## Methods

### Participants

14 normal hearing individuals (9 females, 5 males, average age: 29.1, std.dev: 5.1) took part in the experiment. Initial sample size was determined as 16 participants, 2 of which cancelled their appointments prior to the experiment. Participants gave their informed consent prior to the inclusion in the study and were compensated after the experiment. The study was conducted in accordance with the ethical standards in the Declaration of Helsinki and was approved by the Vastra Gotaland regional ethics committee. Participants completed all the materials individually in a sound-attenuated room.

### Auditory Detection Task

**Figure 1:**
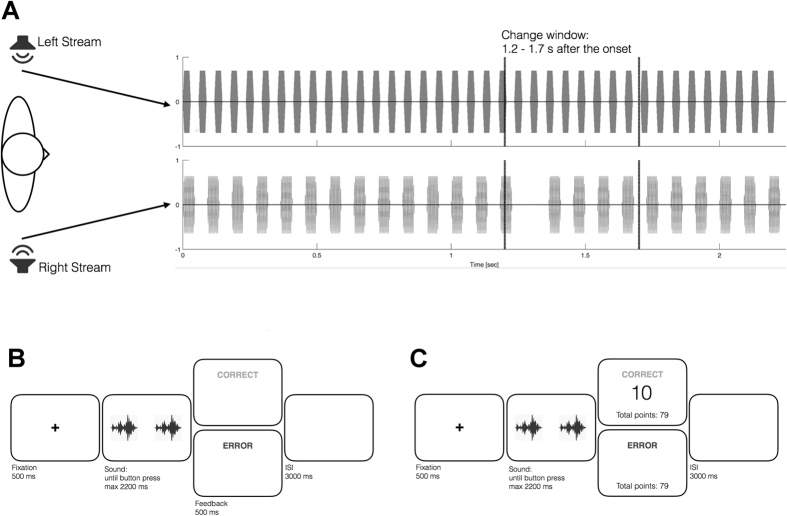
Auditory detection task: (**A**) Two auditory streams were simultaneously presented on participant’s left and right hand side. Streams were amplitude-modulated tones having distinct frequency and modulation rates. In target trials, the target stream contained a 75 ms-long silence period (right-stream in the figure) that was introduced randomly between 1200 and 1700 ms after the stream onset. Participants were instructed to detect the target stream and indicate its location by pressing a respective button. (**B**,**C**) Trials in the pre- and post-reward (**B**), and reward blocks (**C**). Trials stared with a 500-ms fixation period that preceded the simultaneous presentation of auditory streams. For their responses participants received visual feedback. In the reward blocks, they could see their total points and how many points they received for each correct answer (1p or 10p).

During the experiment, participants performed an auditory detection task. In each trial, two sounds were simultaneously presented at two different locations (left and right). Loudspeakers (Genelec-8020) were located on participants’ left and right hand side at a distance of 1.5 meter from the participant. Sounds were amplitude-modulated tones with different frequencies and modulation rates. For amplitude-modulation we used a square-wave whose edges were shaped by 3 ms cosine ramps to avoid clicks. The selection of sounds was based on the research on auditory scene analysis, which concerns the ability of the auditory system to decompose the incoming acoustic input into separate auditory streams^1^. Previous research using square-wave modulated tones found that humans can hear individual auditory streams among the concurrent sounds as long as the pitch and modulation rates were distinct^15^. Each auditory stream formed in this manner sounds like a tone (i.e. tone frequency) with a particular tempo (i.e. modulation rate). In each trial, participants heard two streams with distinct frequencies and modulation rates that were presented simultaneously at separate locations. In target trials, the target stream contained a 75 ms-long silence period that was introduced randomly between 1200 and 1700 ms after the stream onset (Fig. 1A). Participants were instructed to detect the target stream and indicate its location by pressing a respective button (left or right), after which they received visual feedback (correct or error). Any given trial was either a target trial, in which only one target was present, or a catch-trial (i.e. no-target).

#### Reward training

During reward training, participants performed the auditory detection task and earned points for each correct answer. The more points they earned, the more gift cards they received at the end of the experiment. They were instructed to maximize their gains. Participants gained either 1 point (low-reward) or 10 points (high-reward) after each correct answer, and we instructed them that the amount they would receive for a particular correct answer would be determined by a real-time performance assessment algorithm that was not controlled by the experimenter. While in reality, there was a predetermined reward delivery schedule. In this part of the experiment, participants heard the same two sounds in each trial: CS+ and CS−. Correct responses to CS+ were rewarded with a high probability (0.8) of high reward (10p), and a low probability (0.2) of low reward (1p). Whereas correct responses to CS− were associated with a 0.2 probability of earning 10 points and 0.8 probability of earning 1 point. During the reward training, each sound (CS+ and CS−) appeared in each location (left and right) in 50% of the trials. Thus, the spatial location of the target did not predict the amount of reward. In other words, high reward was associated with a particular auditory stream (CS+ or CS−), not with a behavioral response (left or right).

The assignment of CS+ and CS− was carried out in the following manner. We first selected two tone frequencies (250 and 1000 Hz) and modulation rates (11 and 17 Hz). In order to control for the acoustical properties of sounds, we formed all possible combinations of tone frequency and modulation rates, and balanced the assignment of CS+ and CS− for the participants. For instance, if a participant received 250 Hz tone modulated at 11 Hz as CS+, then CS− was 1000 Hz tone modulated at 17 Hz, and that participant only heard those two sounds in each trial during reward training. Thus, for each participant a specific combination of tone frequency and modulation rate predicted high probability of high reward.

Participants completed the reward training of 300 trials (60 were catch-trials) in 6 separate blocks. Each block took approximately 5 minutes. There were two experimental factors in the target trials: target stream (CS+ or CS−), and location of the target stream (left or right), which resulted in four conditions. Each condition was repeated 10 times per block (see Fig. 1C for the timing details of the trials during reward training).

#### Pre- and post-reward tasks

In order to assess the influence of reward learning on auditory attention, participants completed one block before (pre-reward) and one block after the reward training (post-reward). There was no reward delivery during pre- and post-reward blocks, which were precisely the same. Participants simply received visual feedback for their answers (Fig. 1B). We used CS+ and CS− together with a control sound. The control sound was the same for each participant: a 570 Hz tone modulated at 6 Hz. Our main hypothesis here was that if CS+ gains motivational value through reward learning, it would become a more effective distractor when the target is the control stream. Furthermore, the effect of CS− is expected to be in the opposite direction, since during reward-learning successful detection of CS− led to lower rewards while successful suppression of CS− led to higher rewards. Thus, after reward training it would be easier to ignore CS− when the control stream was the target.

Participants completed 60 trials in each block (i.e. pre-reward and post-reward), 12 of which were catch-trials. Target trials had the following experimental factors: stimulus-pair (CS+/CS−, CS+/control, or CS−/control), target stream (one of the two streams in each stimulus-pair), and location of the target stream (left or right). Each of the resulting 12 conditions were repeated 4 times in each block.

### Procedure and Data Analysis

Each participant first completed a training session (30 trials) to become familiar with the experimental task. In this session, they received visual feedback for their responses. After the initial training they completed the pre-reward block. At this point, the procedure for the reward blocks were explained. After the reward blocks, participants were given the gift certificates and instructed that they would complete a post-reward block that would not affect their gains. Once the post-reward block was finished, participants were debriefed and asked whether the reward delivery was fair. Critically, none of the participants were aware of the reward delivery schedule.

Within each block (pre-reward, post-reward, and 6 reward training blocks), individual hit and false-alarm rates were calculated for each experimental condition. Then, we computed perceptual sensitivity (d’) using hit and false-alarm rates. The resulting d’ data were entered into repeated-measures analysis of variance (ANOVA) to test the critical hypotheses. All reported error terms are 95% confidence intervals unless stated otherwise.

## Results

### Reward training

Perceptual sensitivity during reward training were analyzed with an ANOVA with block (reward block 1–6), target (CS+or CS−), and target location (left or right) as within-subject factors. The main effect of block was significant (F(5,65) = 3.555, p = 0.007, η_p_^2^ = 0.22). Pairwise comparisons showed that d’ was lower in the first block, and that it increased in further blocks. Target-location main effect was close to being significant (F(1,13) = 4.143, p = 0.063, η_p_^2^ = 0.24), indicating that participants were slightly more sensitive for the targets located on their left side compared to their right side. Target main effect was not significant.

### Pre- and post-reward blocks

We hypothesized that after reward training CS+ would gain greater motivational value compared to CS−. Hence, when the control stream is the target, CS+ would become a more effective distractor than it was before, while the effect of CS− as a distractor would be in the opposite direction. In order to test this hypothesis, d’ from trials, during which the control stream was the target, were submitted into an ANOVA with block (pre-reward or post-reward), distractor (CS+ or CS−), and target-location (left or right) as within-subject factors. A tendency of the block main effect was found (F(1,13) = 4.458, p = 0.086, η_p_^2^ = 0.21), indicating that the perceptual sensitivity for the control stream was slightly higher in the post-reward (2.89 ± 0.21) compared to pre-reward block (2.76 ± 0.18). Critically, block*distractor interaction was statistically significant (F(1,13) = 8.894, p = 0.011, η_p_^2^ = 0.41), which suggests that the influence of the distractors (CS+ and CS−) changed in the opposite direction after the reward training (Table 1). Mean d’ values indicated that after reward training perceptual sensitivity for the control stream decreased when CS+ was the distractor. However, when CS− was the distractor perceptual sensitivity for the control stream increased after reward training.

#### Additional analyses

In order to be complete, we made other comparisons based on stimulus pairs (CS+/CS−, CS+/control, and CS−/control) in pre- and post-reward blocks. d’ from different stimulus pairs were entered into separate ANOVAs with block (pre-reward or post-reward), target-stream, and target-location as within-subject factors. No effect of reward training was found on CS+/CS− pairs. For both CS+/control and CS−/control pairs, we found a main effect of target-stream (CS+/control: F(1,13) = 9.353, p = 0.009, η_p_^2^ = 0.42; and CS−/control: F(1,13) = 7.813, p = 0.015, η_p_^2^ = 0.38). These main effects showed that d’ for the control stream was higher compared to both CS+ and CS−. We argue that this could be mainly due to the slower modulation rate of the control stream. The breaks seemed to be slightly easier to detect in slow modulating streams (see also, the regression analysis below). Further, block*target interaction was significant for CS−/control trials (F(1,13) = 11.134, p = 0.005, η_p_^2^ = 0.46), which indicated that after reward training perceptual sensitivity for CS− decreased while it increased for the control stream (Table 1).

Finally, we analyzed the effects of acoustical parameters on task performance during pre- and post-reward blocks. We pooled d’ data over participants and experimental conditions, and entered into a regression analysis as the dependent variable. The independent variables were tone frequencies and modulation rates of the respective target and distractor streams. As a result, we found that only the modulation rates of target (std-beta = −0.137, p = 0.025) and distractor (std-beta = 0.179, p = 0.003) had significant contributions to the model (R2 = 0.09, p = 0.001). Results showed that perceptual sensitivity increased with decreasing target modulation rates and increasing distractor modulation rates. Therefore, this finding could account for the higher d’ when the control stream was the target.

## Discussion

The current study aimed to investigate whether reward-dependent attentional learning could bias attentional selection in the auditory domain. We manipulated motivational value via reward-learning, in which selection of a certain sound (CS+) was associated with a high reward, while selection of another sound (CS−) was associated with a low reward. As a result, presentation of CS+ and CS− as distractors affected the task performance differently; that is, after reward-training perceptual sensitivity for a control sound decreased when CS+ was the distractor, while it increased when CS− was the distractor. This finding suggests that through reward-learning CS+ gained motivational value and in turn could bias the attentional selection in its favor. On the other hand, it was easier to ignore CS− after reward-learning. Learning simple stimulus-reward associations may not account for this finding, since that would also predict reduction in performance for trials during which CS− was a distractor (e.g. see refs 6 and 7). Instead, we argue that since successful attentional suppression of CS− during reward trials was associated with high rewards, it became motivational to ignore it. It was suggested that when participants believed that rewards depended on their performance, learning could modulate specific attentional selection and suppression mechanisms acting on the stimulus representations^12^. Our findings are in line with this explanation since participants were told that their performance would determine the rewards. Taken together, these findings show that reward-dependent attentional learning functions in the auditory domain, and can modulate auditory attentional selection and suppression mechanisms in humans.

The current results could also be interpreted in the light of the findings showing that items possessing high motivational and emotional value forms a special group of high-salient stimuli^16^,^17^,^18^. The influence of motivational value on attentional selection has been argued to be distinct from both stimulus-driven and goal-driven modulation of attention^11^. Further, the finding that sounds could acquire value through reward-learning in less than 300 trials, which is around 1000 trials in the visual domain (ref. 19, but also see, ref. 11), points to the adaptive capacity of the auditory system. It was claimed that the auditory system functions as an adaptive-cognitive network specialized in processing acoustic input and that it integrates the information on the behavioral (e.g. emotional and attentional) state of the organism to its processing^20^. Conditioning studies on animals found that frequency selectivity of the cells in the auditory system can change to enhance their responses to the CS+ frequency at the expense of other frequencies. This receptive field plasticity was observed both in the primary auditory cortex (A1) and in other structures in the auditory system^21^. Receptive field plasticity due to associative learning in A1 is highly specific to the CS+ frequency, develops rapidly in as few as five trials, shows long-term retention (can endure up to 8 weeks after a 30-trial conditioning session), and continues to develop (for a detailed account, see ref. 20). Also, this was shown in both positive and negative affective contexts, and in several species including humans. Therefore, our findings –in line with the adaptive capacity of the auditory system– indicate that emotional or motivational information in auditory stimuli leads to biases in auditory processing and adapts the system to be more attentive and tuned to significant events^22^.

The present study is the first to provide clear behavioral evidence that reward-learning could modify auditory attentional selection and suppression mechanisms in humans. Research on reward-learning in the auditory domain is scarce. To our knowledge, two studies used auditory reward-learning to form simple stimulus-reward associations; and tested their impact on visual perception^23^ and attention^24^. Pooersmaeili and colleagues^23^ found that high-reward associated sounds increased visual sensitivity in an orientation discrimination task, while Anderson^24^ found that high-reward associated sounds interfered with the performance in a visual attention task. These seemingly contradicting results indicate that it is currently unknown how exactly the value representations in auditory domain would affect visual processes. In the current study, on the other hand, we focused on the impact of reward-learning on auditory attentional selection, and the task we designed reflected the attentional mechanisms during sound perception. As was introduced before, the auditory system decomposes the incoming input into separate information streams^1^, which then compete for attentional resources^3^. Hence, the current finding that reward-learning could modulate attentional mechanisms during attentional competition contributes to the organizing principles of auditory processing.

Further, previous research has shown that emotional salience of sounds could influence attention and perception (e.g. refs 4, 5 and 25). However, those mainly concerned emotionally negative stimuli and their influences on auditory attention with findings indicating that negative stimuli can bias the attentional selection. While, in the present study we show that positive value of sounds can also modulate attentional selection in the auditory domain. Future studies should focus on the specific mechanisms associated with reward-learning in the auditory domain. For instance, how different stimulus features and perceptual contexts (e.g. ref. 26) influence reward-learning; and whether the effects are generalizable to different attention tasks. Further research should also investigate whether rewards can modulate auditory spatial attention as well (for findings in the visual domain, see refs 27 and 28).

## Additional Information

**How to cite this article**: Asutay, E. and Västfjäll, D. Auditory attentional selection is biased by reward cues. *Sci. Rep.* **6**, 36989; doi: 10.1038/srep36989 (2016).

**Publisher’s note**: Springer Nature remains neutral with regard to jurisdictional claims in published maps and institutional affiliations.

## Figures and Tables

**Table 1:** Mean d’ for all the targets in the CS+/control, CS−/control pairs during the pre-reward and post-reward blocks (95CI are indicated in the parentheses).

Stimulus Pair	Target Stream	Pre-Reward	Post-Reward
CS+/Control	CS+	2.08 (0.43)	2.19 (0.61)
	Control	3.03 (0.22)	2.81 (0.31)
CS−/Control	CS−	2.33 (0.37)	1.98 (0.58)
	Control	2.48 (0.32)	2.98 (0.25)
